# Surgical Pathology and Therapeutics

**Published:** 1900-09

**Authors:** 


SURGICAL PATHOLOGY AND THERAPEUTICS. By JOHN COLLINS WARREN, M. D., LL. D., Professor of Surgery in Harvard University; Surgeon to the Massachusetts General Hospital. Illustrated. Second edition, with an appendix, containing an enumeration of the Scientific Aids to Surgical Diagnosis, together with a series of Sections on Regional Bacteriology. Philadelphia: W. B. Saunders. 1900.

The title page and preface of the second edition of Warren's Surgical pathology and therapeutics draws attention to the appendix in which important changes are included, replacing the old appendix and the chapter on antiseptic surgery.

The excellence of the first edition, the exhausting of the supply, thus putting the work out of print, justify the second edition. The appendix is in the main a review of magazine articles with so little of positive information that it should not be offered as a justification for a second edition of the work. The author states some conclusions of value in the brief discussion he devotes to blood examination. In the paragraphs on the urine, the feces, immediate microscopical examination of sections of tumor during operations, and the Röntgen rays, a most brief and guarded summing up of recent pathological work is made. The chief value of the appendix is to be found in the deductions drawn from bacteriological work done on the bacteria of the skin and orifices of the body. The application of our knowledge in these fields to wound treatment and healing deserves commendation.

A clearer and more positive statement of the difference between hyperemia or non-infective, inflammation and inflammation as a process due to infection should have been made in the light of what has been done in the last few years in this field. To leave the old confusing ideas of the first edition on this subject is inexcusable.

The work is chiefly valuable to the student and practitioner interested in the newer pathology, because it so well expresses the thought of men of the school of five years ago. E. A. S.

